# Supplementary material for: Sub-1 Volt and high-bandwidth visible to near-infrared electro-optic modulators
Source: Nat Commun. 2023 Mar 27;14:1496. doi: 10.1038/s41467-023-36870-w (PMC10042872; doi:10.1038/s41467-023-36870-w)
Supplement: Supplementary file 1 — Supplementary Information [file 41467_2023_36870_MOESM1_ESM.pdf]

Supplementary Information File: Sub-1 Volt and High-Bandwidth Visible to Near-Infrared Electro-Optic Modulators

Dylan Renaud[1*†], Daniel Rimoli Assumpcao[1†], Graham Joe[1], Amirhassan Shams-Ansari[1], Di Zhu[1,2], Yaowen Hu[1,3], Neil Sinclair[1,4] and Marko Loncar[1*]

[1]John A. Paulson School of Engineering and Applied Sciences, Harvard University, Cambridge, 02139, MA, United States.
[2]Institute of Materials Research and Engineering, Agency for Science, Technology and Research (A*STAR), 138634, Singapore.
[3]Department of Physics, Harvard University, Cambridge, 02139, MA, USA.
[4]Division of Physics, Mathematics and Astronomy, and Alliance for Quantum Technologies (AQT), Harvard University, Cambridge, 02139, MA, USA.

*Corresponding author(s). E-mail(s): renaud@g.harvard.edu; loncar@g.harvard.edu;
†These authors contributed equally to this work.

Keywords: Integrated Photonics, Frequency Comb, Frequency Shifting

Inventory of Supplemental Information

1. Supplementary Figure 1
2. Supplementary Figure 2
3. Supplementary Table 1
4. Supplementary Table 2
5. Supplementary References

Supplementary Figure 1

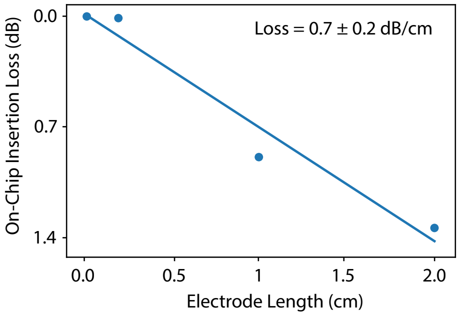

Supplementary Fig. 1: **Modulator loss via cutback method.** On-chip transmission loss as a function of metal electrode length. Data is plotted relative to the linear fit y-intercept. A linear fit yields a loss of 0.7 dB/cm, with $\pm$ 0.2 error arising from the fiber-to-chip coupling efficiency variation.

We extract the modulator on-chip loss by fabricating an array of 3 μm gap modulators of varying electrode length, measuring the transmission as a function of length over the 710-740 nm range, and then fitting the result. The device on-chip transmission loss and resultant linear fit are shown in supplementary Fig. 1. From this, we obtain an on-chip loss of 0.7 $\pm$ 0.2 dB/cm. The error is derived from the variation of the fiber-to-chip coupling efficiency. The lensed fiber-to-chip coupling efficiency is measured to be approximately 7 dB/facet, and arises from the large modal mismatch between the lensed fiber and rib waveguide modes. Thus, the total device insertion loss of our 3 μm gap, 1 cm long modulator is $\sim$ 15 dB.

Supplementary Figure 2

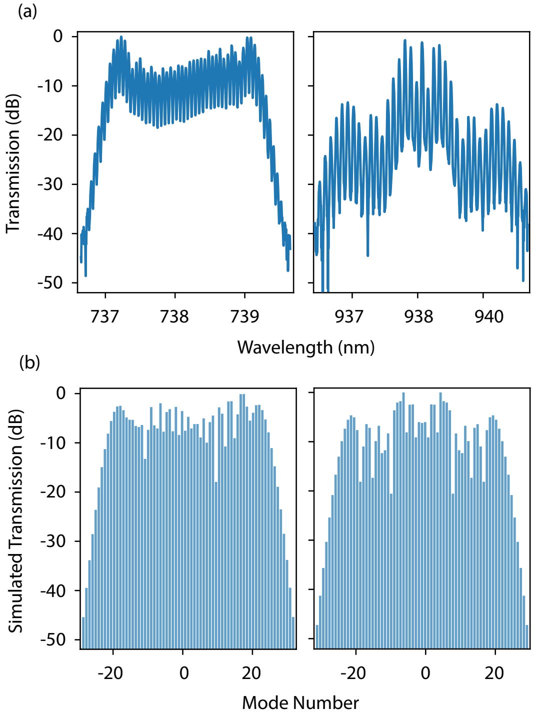

Supplementary Fig. 2: **Envelope asymmetry and comb nonuniformity in VNIR electro-optic frequency combs. a,** VNIR combs generated at $\lambda = 638$ (left) and $\lambda = 938$ nm (right) displaying asymmetry and non-uniformity.**b,** Both the asymmetric (left) and non-uniform (right) phenomenon can be reproduced theoretically by considering a combination of spatial modes being modulated inside of the waveguide.

The spectral asymmetry of the shorter wavelength combs can be explained by considering the impurity of the spatial mode in the waveguide. For example, if the input mode is a combination of fundamental TE and TM modes, both modes will experience a phase modulation and the generated frequency comb of each mode will interference with the other. The two modes will first have an optical phase difference of $\Delta\psi_0$ which contributes to a global phase difference for all the frequency comb lines. More importantly, due to the difference of group velocity dispersion of the two modes, the microwave signal that each mode experienced will have a phase difference of $\Delta\psi_{MW}$. As the comb line phase caused by the microwave phase will accumulate when light spreads over the frequency domain, this $\Delta\psi_{MW}$ causes the spectral asymmetry. To model

this effect, we consider that the field that passes through a conventional phase modulator, which can be expressed as

$$E_1 = A_0 e^{i\omega_L t} e^{i\beta_1 \sin\omega_{MW} t} = \sum_{q=-\infty}^{\infty} A_0 J_q(\beta_1) e^{iq\omega_{MW} t} e^{i\omega_L t} \tag{1}$$

where β_1 is the modulation index for fundamental TE mode, $J_q(\beta_1)$ represents q-th order Bessel function, q is the Bessel function mode number, ω_{MW} is the microwave driving frequency, ω_L is the laser frequency, and A_0 is a global amplitude parameter for the fields.

The output optical fields can be derived as the following form:

$$E_1 = A_0 e^{i\omega_L t} e^{i\beta_1 \sin\omega_{MW} t} + A_0 e^{i\omega_L t} e^{i\Delta\psi_0} e^{i\beta_1 \sin\omega_{MW} t + \Delta\psi_{MW}} = \sum_{q=-\infty}^{\infty} A_0 (J_q(\beta_1) + e^{i\psi_0} e^{iq\Delta\psi_{MW}} J_q(\beta_1)) e^{iq\omega_{MW} t} e^{i\omega_L t} \tag{2}$$

in which the β_1 and β_2 are the modulation indexes for fundamental TE mode and another impurity mode, respectively. In this equation, the term $e^{iq\Delta\psi_{\mathrm{MW}}}$ provides varied phase differences between the two modes for each frequency comb line. Therefore different interferences occur at different frequencies, leading to the envelop asymmetry. The simulated results using this equation are shown in supplementary Fig. 2.

Supplementary Table 1

Type	Half-wave Voltage (V_π)	3-dB Electro-optic Bandwidth	Ref
AlN	12.9 V	0.14 GHz	[1]
AlN	50-65 V	0.12 GHz	[2]
TFLN	4.2 V	2.7 GHz	[3]
TFLN	8 V	10 GHz†	[4]
TFLN	3.3 V	25 GHz†	[5]
TFLN	4.5 V	6.5	[6]
Bulk LN	2.5 V	12 GHz	[7]
Bulk LN	3.5 V	25 GHz	[8]
Bulk LN	6.5 V	40 GHz	[9]
Polymer	0.52 V	1 KHz†	[10]
This Work	0.42-0.85 V	35 GHz	-

Supplementary Table 1: **Comparison of VNIR modulator platforms.** Comparison of various VNIR modulator's voltage and bandwidth performance discussed in main text Fig. 2d.

†: Measured bandwidth limited by equipment.

Supplementary Table 2

	This Work	Ref [3]	Ref [4]	Ref [5]	Ref [6]
Wavelength (nm)	532-938	780	850	532	940
V_π (V)	0.42-0.85	4.2	8	3.3	4.5
3-dB B.W (GHz)	35	2.7	10†	25†	6.5
On-Chip Loss (dB)	0.7 ± 0.2	N/A*	N/A*	7.3	0.8 ± 0.3
E.R. (dB)	21-25	19.4-27	15-30	23	21

Supplementary Table 2: **Comparison of TFLN VNIR modulators.** Comparison of VNIR TFLN modulator V_π, 3-dB EO bandwidth, on-chip loss, and extinction ratio (E.R.) performance.

†: Measured bandwidth limited by equipment.
*: Manuscript does not report a measured modulator loss.

Supplementary table 1 provides detailed half-wave voltages and 3-dB electro-optic bandwidths of VNIR AlN, thin-film LN (TFLN), polymer, and commercial LN modulators. The bandwidths with asterisks denote devices for which the measured bandwidth was limited by available equipment. We also compare more detailed performance metrics (V_π, bandwidth, on-chip loss, extinction ratio) between this work and other VNIR TFLN modulators in supplementary table 2.

Supplementary References

[1] Zhu, S. *et al.* Aluminum nitride ultralow loss waveguides and push-pull electro-optic modulators for near infrared and visible integrated photonics. *2019 Optical Fiber Communications Conference and Exhibition (OFC)* 1–3 (2019) .

[2] Dong, M. *et al.* High-speed programmable photonic circuits in a cryogenically compatible, visible–near-infrared 200-mm cmos architecture. *Nature Photonics* **16**, 59–65 (2022). https://doi.org/10.1038/s41566-021-00903-x .

[3] Celik, O. T. *et al.* High-bandwidth CMOS-voltage-level electro-optic modulation of 780 nm light in thin-film lithium niobate. *Optics Express* **30** (13), 23177 (2022). URL https://doi.org/10.13642Foe.460119. https://doi.org/10.1364/oe.460119 .

[4] Desiatov, B., Shams-Ansari, A., Zhang, M., Wang, C. & Lončar, M. Ultra-low-loss integrated visible photonics using thin-film lithium niobate. *Optica* **6** (3), 380–384 (2019). URL http://opg.optica.org/optica/abstract.cfm?URI=optica-6-3-380. https://doi.org/10.1364/OPTICA.6.000380 .

[5] Li, C. *et al.* High modulation efficiency and large bandwidth thin-film lithium niobate modulator for visible light. *Optics Express* **30** (20), 36394–36402 (2022) .

[6] Sund, P. I. *et al.* High-speed thin-film lithium niobate quantum processor driven by a solid-state quantum emitter. *arXiv preprint arXiv:2211.05703* (2022) .

[7] Kg-pm series 780nm series eo modulator. http://www.conquer-oc.com/en/detail-92-95-504.html (Accessed: 11/2022).

[8] Ixblue nir-mx800-ln-20-00-p-p-fa-fa. https://www.ixblue.com/north-america/store/nir-mx800-ln-20-00-p-p-fa-fa/ (Accessed: 11/2022).

[9] Optilab 785 nm, 40 ghz intensity modulator. https://www.optilab.com/products/785-nm-40-ghz-intensity-modulator-pm-output (Accessed: 11/2022).

[10] Kamada, S. *et al.* Superiorly low half-wave voltage electro-optic polymer modulator for visible photonics. *Optics Express* **30** (11), 19771–19780 (2022) .
